# A Review: Sample Preparation and Chromatographic Technologies for Detection of Aflatoxins in Foods

**DOI:** 10.3390/toxins12090539

**Published:** 2020-08-21

**Authors:** Kai Zhang, Kaushik Banerjee

**Affiliations:** 1 US Food and Drug Administration/Center for Food Safety and Applied Nutrition, 5001 Campus Drive, College Park, MD 20740, USA; 2 National Reference Laboratory, ICAR-National Research Centre for Grapes, Pune 412307, India; kbgrape@yahoo.com

**Keywords:** aflatoxins, chromatography, sample preparation

## Abstract

As a class of mycotoxins with regulatory and public health significance, aflatoxins (e.g., aflatoxin B_1_, B_2_, G_1_ and G_2_) have attracted unparalleled attention from government, academia and industry due to their chronic and acute toxicity. Aflatoxins are secondary metabolites of various *Aspergillus* species, which are ubiquitous in the environment and can grow on a variety of crops whereby accumulation is impacted by climate influences. Consumption of foods and feeds contaminated by aflatoxins are hazardous to human and animal health, hence the detection and quantification of aflatoxins in foods and feeds is a priority from the viewpoint of food safety. Since the first purification and identification of aflatoxins from feeds in the 1960s, there have been continuous efforts to develop sensitive and rapid methods for the determination of aflatoxins. This review aims to provide a comprehensive overview on advances in aflatoxins analysis and highlights the importance of sample pretreatments, homogenization and various cleanup strategies used in the determination of aflatoxins. The use of liquid-liquid extraction (LLE), supercritical fluid extraction (SFE), solid phase extraction (SPE) and immunoaffinity column clean-up (IAC) and dilute and shoot for enhancing extraction efficiency and clean-up are discussed. Furthermore, the analytical techniques such as gas chromatography (GC), liquid chromatography (LC), mass spectrometry (MS), capillary electrophoresis (CE) and thin-layer chromatography (TLC) are compared in terms of identification, quantitation and throughput. Lastly, with the emergence of new techniques, the review culminates with prospects of promising technologies for aflatoxin analysis in the foreseeable future.

## 1. Introduction

Aflatoxins are a class of toxic metabolites generated by certain fungi that are commonly found in the environment. *Aspergillus flavus* and *Aspergillus parasiticus* are the dominant fungal species that produce aflatoxins under drought, warm and humid conditions [1,2,3]. In the 1960s, Turkey “X” disease resulted in the death of over 100,000 turkeys and other poultry in England [4,5,6,7]. The outbreak, associated with contaminated feed, led to the identification of aflatoxins and commenced research on aflatoxin toxicity. Since then aflatoxin contamination has been gradually recognized as a threat to global food safety and trade [8,9,10,11]. Continuous and collaborative efforts from government, academia and industry have been dedicated to understanding and mitigating the impact of aflatoxins on public health and economy [12,13,14].

Aflatoxins are well known to contaminate crops in the field and during storage [15]. Commonly consumed food commodities such as cereals, peanuts and dairy products are a major concern for aflatoxin contamination because current agricultural practices and food processing techniques cannot completely remove aflatoxins [1,16,17]. Consequently, aflatoxins can enter the food chain and give rise to health and trade related issues [18,19,20]. Among various types of aflatoxins, the predominant ones—aflatoxin B_1_, B_2_, G_1_ and G_2_ and aflatoxin M_1_, a hydroxylated metabolite of aflatoxin B_1_—can pose a severe threat to human and animal health via dietary exposure.

Toxicity of aflatoxins is well–documented. As reported by the International Agency for Research on Cancer (IARC), aflatoxins are carcinogenic and hepatotoxic to humans. Aflatoxin B_1_, B_2_, G_1_ and G_2_ are classified as human carcinogens (group 1B). Aflatoxin B_1_ is the most potent carcinogen within the group. Although aflatoxin M_1_ is less hepatotoxic and immunotoxic than its parent compound, aflatoxin B_1_, it is stable under pasteurization temperature used to process milk. It is classified as a possible human carcinogen (group 2B) by the IARC. [21]. In response, regulatory bodies worldwide have set regulatory levels for aflatoxins, especially for aflatoxin B_1_ and aflatoxin M_1_, in various commodities [22]. For example, in peanuts, regulatory levels range from 2 µg/kg for aflatoxin B_1_ and 4 µg/kg for total aflatoxins (sum of aflatoxin B_1_ + B_2_ + G_1_ + G_2_) by the European Union [23] to 20 µg/kg for total aflatoxins by the United States Food and Drug Administration [24]. Similarly, the Food Safety and Standards Authority of India [25] has set the limit of total aflatoxins at 10 µg/kg for ready-to-eat nuts and 15 µg/kg for processed nuts. In the US, the action level of aflatoxin M_1_ in raw milk is 0.5 µg/kg, while in the EU the maximum level of aflatoxin M_1_ in milk is 0.05 µg/kg and 0.025 µg/kg in infant formulas and dietary foods for special medical purposes intended specifically for infants. Large variability among these regulatory levels exists because of the requirements (feasibility and economic impact) and approaches to risk analysis are defined in national/regional legislation [26].

**Figure 1:**
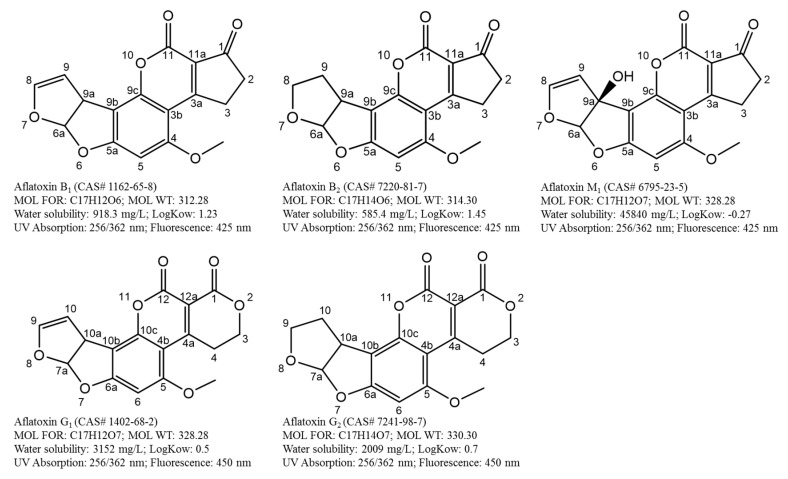
Important physicochemical properties of aflatoxin B_1_, B_2_, G_1_, G_2_ and M_1_.

To comply with established regulatory levels and ensure food and feed are safe for consumption and trade, development of analytical methods that are suitable for monitoring and surveillance of aflatoxins are critical. A well-established aflatoxin method is based on a thorough understanding of the target analytes (polarity, water solubility, thermostability, partitioning coefficient, pKa, etc.), food composition (e.g., fat, water and sugar content), extraction conditions, sample clean-up and instrumentation. Figure 1 shows the important physicochemical properties of aflatoxins B_1_, B_2_, G_1_, G_2_ and M_1_.

Development of sample preparation and detection techniques has been influenced by these properties. For example, selection of extraction solvents should be based on the polarity and solubility of aflatoxins. Detection and quantitation of aflatoxins could be achieved using their UV and fluorescence spectrum. Molecular ions and structurally specific fragments of aflatoxins are often used to achieve unambiguous identification in mass spectrometry analysis. Over the years, numerous methods have been developed to address challenges including heterogeneous distribution, difficulties in cleanup strategies for diverse matrices, laborious steps during extraction and matrix effects. By retrospectively comparing different techniques and evaluating their potentials and limitations, we can understand and appreciate how important discoveries in the past have led to our current aflatoxin analysis procedures and the challenges we need to address now and the directions we want to take in the future. So, it would be worthwhile to provide a comprehensive summary of developed sample preparation and analytical technologies for the determination of aflatoxins. In brief, this review not only outlines the significant advances in sample preparation, and analytical strategies in the area of aflatoxin analysis in a wide range of food matrices, namely cereals, peanut, spices and dairy products, but also illuminates existing and emerging challenges that should be addressed presently and in the foreseeable future from a practical standpoint.

## 2. Sample Preparation

Aflatoxin samples are generally collected from the field following predefined sampling plans [27,28,29]. After sample collection, determination of aflatoxins relies on how samples are homogenized, followed by the removal of test portions (analytical samples) to ensure representative qualitative and quantitative analytical data can be generated to support risk assessment, exposure studies and compliance activities. Furthermore, due to the limitation of available detection technologies, the majority of foods that are prone to mycotoxin contamination cannot be directly analyzed for aflatoxins without extraction and clean-up, a fact that has been incentivizing researcher to use different extraction and clean-up methods to separate aflatoxins from matrix components. The purpose of sample preparation is to minimize the impact of heterogeneous distribution of aflatoxins in various food commodities [30,31,32], selectively enrich for aflatoxins based on their physicochemical properties and reduce bulk background matrix to make final extracts more suitable for instrumental analysis.

### 2.1. Sample Homogenization—Dry, Wet and Cryogenic Grinding

In this section, we offer a glance at various homogenization techniques, namely, dry, wet and cryogenic grinding techniques. Aflatoxins have been found in a variety of foods, including peanuts, tree nuts, oil seeds, cereal grains and miscellaneous commodities such as dried figs, milk, cheese and spices. Most samples such as peanuts and in-shell nuts can be directly ground and blended until sample particles are small enough to meet a predefined degree of fineness (e.g., pass a No. 20 sieve) [33], minimizing the impact of heterogeneous distribution of aflatoxins on quantitation. Depending on the size of a sample and available milling devices, the sample could be first coarse ground to pass through a bigger sieve (e.g., No. 8 or 10). Sub-samples are then removed and blended for fine grinding to pass through a No. 20 sieve [34]. Alternatively, the entire coarse ground sample is finely ground before the test portion or analytical sample is removed [33,35]. Dried commodities (e.g., corn kernels) can be ground with the addition of water [36]. Similarly, green coffee beans or dried fruits are cryogenically ground using dry ice in place of water. [37].

#### 2.1.1. Dry Grinding

Many studies have demonstrated the efficiency of dry grinding procedures for different matrices, aiming at sufficient practical size reduction (e.g., passing through #20 sieve) and uniform distribution of contaminated portions via blending and mixing. For instance, using dry grinding, Asghar et al., 2014 [38] pulverized brown rice samples into the size of less than 1 mm and sieved samples to obtain a homogenous sample particle size. The same strategy of grinding (and sieving) was applied in red chili [39], and dry date [40] for the determination of aflatoxin B_1_. Dhanshetty et al., 2019 [41] also demonstrated the use of dry grinding for a range of cereal matrices providing satisfactory homogeneity and recoveries of detected aflatoxins (84–106%). In another study, Zahra et al. [42], who performed dry grinding as per the AOAC method [34] for various spices (e.g., black pepper and red chilies) and dry fruits (e.g., dried apricots and figs). Spanjer et al., 2008 [43] successfully used dry milling to process peanut, pistachio, wheat, maize, cornflakes, raisins and figs for the analysis of multiple mycotoxins including aflatoxin B_1_. However, due to capacity limitations of their device, the drying milling protocol was only developed for matrices with sample sizes less than 1.5 kg.

Numerous heavy-duty and batch-type milling and mixing devices (e.g., Hobart VCM, Waring Blendor and Polytron) are commercially available and have been tested in cereal grains, tree nuts, botanicals and feeds [44,45,46,47]. Although, dry grinding has been widely used by mycotoxin labs, when sample sizes are large or food products contain high sugar or fat content, dry grinding could suffer clogging or buttering due to excessive heat generated by fast moving blades. In such situations, sample grinding results in insufficient homogenization (e.g., particle sizes are too large) [48]. Therefore, despite its laborious and time-consuming nature, wet grinding can serve as an alternative milling approach, providing better homogenization with smaller particle sizes and more uniform distribution of aflatoxins.

#### 2.1.2. Wet Grinding

Wet grinding is performed by mixing and blending samples and water or extraction solvents together. Careful efforts should be invested to optimize solvent/sample ratios, which is critical to homogeneity and extraction efficiency. Velasco and Morris [36] evaluated wet grinding procedures for aflatoxins analysis in peanut, whole seeds, peanut and cottonseed meals and copra. Using a matrix-dependent sample to water ratios (e.g., 1:1.5 for peanut butter, 1:2.25 for whole seeds, 1:4 for commercial corn and cottonseed meals and 1:5 for copra), the authors demonstrated that making slurries of aflatoxin samples led to more uniform distribution of particles and thus better reproducibility among test portions than conventional dry millings. In order to improve extraction efficiency of aflatoxin from raw peanuts, Whitaker et al. [49,50] conducted a series of tedious studies by testing 49 different combinations of methanol concentrations and solvent/peanut ratios. It was concluded that a combination of 60% methanol and a 10.8 mL solvent/g peanuts ratio might be used to achieve optimal extraction efficiency for aflatoxins.

The distribution of aflatoxins in heterogeneous test matrices was evaluated by Scholten and Spanjer (1996) using pistachio samples in the shell [51]. In this work, pistachios samples (up to 3 kg per sample) were homogenized with water and sodium chloride (150 mL tap water and 4 g NaCl/100 g sample) and blended for 30–45 min. The formation of a wet slurry provided a homogenous matrix, which is difficult to achieve for pistachios using dry milling. Another benefit of this procedure was to avoid manually shelling pistachios, a painstaking effort to prepare tree nuts samples with shells for dry milling. Spanjer et al., 2006 [48] reported the milling and homogenization processes for cocoa, green coffee, almonds and pistachio, where the authors demonstrated the effect of dry milling and wet milling. The homogenization process was evaluated in terms of the analytical results, coefficients of variation for different mills, sample and subsample sizes and particle size distributions. The study concluded that the coefficients of variation were higher for dry milling than those for slurry mixing. As an extension of the study in 2006, Spanjer et al. [43] applied the developed wet milling method to matrices with sample sizes greater than 1.5 kg to ensure homogeneity of the final samples for LC-MS/MS analysis. In a similar study, Schatzki et al., 2003 [52] compared the slurry mixing method to dry grinding procedures in pistachios. The results demonstrated that after mixing the pistachio sample with water (sample/water mass ratio, 1/2), the resulting coefficient of variation is less than half that of the dry grinding. Interestingly, the study also suggested that wet grinding would enhance the efficiency of extraction of aflatoxins from smaller particles. Therefore, aflatoxin concentrations determined in some matrices using wet grinding could be higher than those done by dry milling. Similar observations were reported in other studies [36,53], underlying the effects of sample sizes, matrix compositions, as well as grinding devices/conditions should be carefully evaluated. In a recent study, Kumphanda et al. [54] demonstrated the analytical variances associated with testing a 25.0 g slurry (1:1, sample:water), 50.0 g dry grind and 12.5 g dry grind sample portion in the ratio of 1:5:15, suggesting that the wet grinding could provide improved homogeneity for cereals.

In recent years, LC-MS-based mycotoxin analysis has been increasingly used. Due to its superior sensitivity, test portions (0.5–5 g) for LC-MS analysis are much smaller than those of LC-Ultraviolet/Fluorescence detection (LC-UV/FLD) (25–50 g). To ensure test portions would give representative information of the sample, Zhang et al., 2017 [55] developed a secondary fine-milling procedure for LC-MS/MS-based multimycotoxin analysis. A subsample (25 g) of corn or wheat flour was taken and mixed with water (sample to water mass ratio, 1/1) and 2 g of the resulting slurry was taken for extraction and LC-MS/MS analysis. Using this procedure, the impact of potential homogeneity concerns is minimized. Furthermore, this approach builds on the selection of a subsample size (e.g., 25–50 g) previously shown to be representative of the initial sample, an approach that would allow scaling to small analytical test portions. An additional benefit of small test portions is the significant reduction in solvents used and waste generated, making sample analysis more cost-effective.

#### 2.1.3. Cryogenic Grinding

In aflatoxin determination, cryogenic milling (cryo-milling) has potential applications as it permits heat sensitive materials to be effectively ground into very small particle sizes [56]. The utility of cryogenic milling has been demonstrated for predominantly fatty food and feed (e.g., groundnuts and derived products, almonds, pistachios, Brazil nuts, maize, rice, figs, cotton seed and spices) matrices [57]. Furthermore, cryogenic milling also retains the nature of the matrix more than dry or wet milling. This is very important for the preparation of proficiency test samples and reference materials [58,59]. The excessive heat generated by dry milling or the addition of water in wet milling modifies the physical composition of starting materials, compromising of the quality of the final reference material. For example, to prepare starting materials for a National Institute of Standards and Technology (NIST) standard reference material (SRM 1565), Phillips et al., 2019 [59] used cryogenic milling to homogenize and blend 70 kg of blank corn and 14 kg of incurred corn. The analysis of particle size distribution shows that in the final blank and incurred corn, the average particle sizes are 588 µm with a standard deviation of 17 µm (*n* = 10) and 676 µm with a standard deviation of 92 µm (*n* = 10), respectively. Due to the homogeneity of the material, test portions ranging from 1 to 5 g would be enough to ensure analytical results are in a good agreement with certified values. Currently, to ensure homogeneity of test samples and accommodate a wide range of analytical procedures used by participating labs, many proficiency test organizers use cryogenic milling to prepare mycotoxin materials [58].

Cryogenic milling requires special milling devices and dry ice or liquid nitrogen as the cooling agent. To achieve better grinding, samples are preferred to be stored in freezers overnight before grinding. After grinding, samples cannot be directly extracted to allow sufficient time for CO_2_ to sublime. Prior to extraction or weighing, samples also need to equilibrate to room temperature otherwise moisture in the air could condense on the samples. Though dry milling has been preferred by many labs due to it being easy for use, it is noted that wet or cryogenic grinding could be successfully applied to accommodate food matrices that are not suited for dry milling. The heat generated during dry grinding resulted in the formation of clogging, which may hinder the formation of homogenous particle size across the matrix. Whereas, in the case of wet or cryogenic grinding, the addition of water and dry ice to the matrix allows the formation of particles with smaller and more uniform sizes and therefore homogenous distribution of aflatoxins. Wet grinding appears superior in providing a uniform particle size distribution and a homogeneous matrix, but it has certain disadvantages, which includes difficulties in cleaning the grinding apparatus. Regardless of grinding techniques used, one of the continuing challenges is to characterize the correlation between homogeneity of the final sample and grinding conditions (devices, milling time, sample type, sample size, subsample size and test portion size). Use of sample sizes too small can lead to biased analysis of aflatoxins with high variability. On the other hand, using unnecessary large sample sizes could increase operation cost and impact throughput when a large number of samples need to be processed. Therefore, in the course of routine sample analysis or evaluation of new homogenization devices/protocols, a homogeneity test and quality control should be performed to ensure that the homogenization process could meet the predefined degree of fineness in the final sample. In some situations, conventional homogeneity protocols may be cumbersome [60], so it is important to explore and adopt existing technologies such as light scattering to monitor the homogeneity of processed samples in an efficient manner [59,61].

Diversity of food commodities that need to be analyzed for aflatoxins makes it impossible to establish a universal milling protocol for all sample matrices. Instead, food commodities can be classified according to composition complexity including fat, protein, water, pigments and other interferences. Products that are similar in composition can often be homogenized by the same or slightly modified methods. Based on the class of the product, past experience and the literature record of milling applications, the most appropriate milling practice can be selected and developed in a practical manner.

### 2.2. Extraction

The diverse nature of aflatoxin sample matrices demands the use of a combination of extraction and clean-up techniques in treating a sample prior to instrumental analysis. To achieve satisfactory recoveries of aflatoxins, extraction needs to be applied to samples first, followed by clean-up, if necessary. For dry matrices, hydration is generally required to wet and swell samples, ensuring efficient extraction and separation of aflatoxins absorbed within the sample. For fatty matrices, additional defatting steps are critical to eliminate co-extracted fatty interferences (e.g., lipid and cholesterol). In this section, commonly used extraction solvents (e.g., acetonitrile, methanol, chloroform and hexane) and techniques are reviewed and compared for their applications in aflatoxin analysis.

#### 2.2.1. Liquid-Liquid Extraction (Partitioning)

Aflatoxins are more soluble in polar organic solvents (chloroform, methanol, acetonitrile, etc.) than in non-polar organic solvents such as hexane [62,63] so liquid-liquid extraction (partitioning) has been widely used to extract and separate aflatoxins from a wide variety of food matrices using immiscible solvent systems containing two or more solvents. In the 1960s and early 1970s, without other extraction and clean-up techniques available, aflatoxin analysis was mainly conducted by liquid-liquid extraction coupled with paper chromatography or thin layer chromatography (TLC). Aflatoxins samples were generally extracted using chloroform, methanol or a mixture of different polar organic solvents, followed by an evaporation step to reduce the volume and concentrate aflatoxins in the extract. The remaining residues were reconstituted and defatted by partitioning between a polar and a non-polar organic solvent (e.g., methanol and petroleum ether). The aflatoxins in the polar phase were processed using TLC. Aflatoxin identification and quantitation was achieved by a comparison of retention factor (Rf) and the intensity of fluorescence of the sample to standards on the TLC plate [64]. For example, in a method developed by Parker and Melnick [65], three consecutive liquid-liquid extraction procedures were used to purify aflatoxins from oil samples. In the first extraction, oil samples were extracted three times using methanol/water, partitioning aflatoxins into the methanol phase. In the second extraction, the combined methanol extracts were defatted using hexane (200 mL) to remove coextracted fatty components from the methanol phase. After evaporation of the aqueous methanol solution to 50 mL, the third liquid-liquid partitioning was performed using chloroform. The resulting extracts were analyzed using a TLC procedure. In order to remove lipid and pigment interferences, Pons and Goldblatt [66] used a series of liquid-liquid extractions to extract aflatoxins from cottonseed samples, which were first extracted using acetone and water (70/30, *v*/*v*). The pigment in the acetone/water phase was precipitated as insoluble lead salt. Aflatoxins were then partitioned into chloroform from the acetone/water phase by repetitive liquid-liquid extractions prior to TLC analysis.

Since the late 1960s, liquid-liquid extraction has been gradually coupled with other developed clean-up techniques (e.g., silica gel chromatography) for aflatoxin analysis [67,68,69,70,71,72]. For example, to determine aflatoxins in peanut butter, Tarter et al. [71] used methanol to extract samples, followed by partitioning fatty interference into hexane. The remaining aflatoxins in the aqueous methanol phase were partitioned into chloroform. Then the chloroform extracts were further purified using an in-house developed silica gel chromatography method. Two laboratories tested the method in eight different matrices, including peanut butter, and achieved consistent results. The mean recoveries of spiked aflatoxins (15–34 ng/g) ranged from 86 to 94% with coefficients of variation less than 7% for spiked samples and 15% for naturally contaminated samples.

Sizoo and van Egmond [72] used liquid-liquid extractions to prepare samples for a total diet study, focusing on aflatoxin B_1_ and M_1_, and ochratoxin A. The samples (10 g each) were first extracted with chloroform (100 mL), followed by filtration and evaporation of 50 mL of the extracts. The remaining residue was purified by a liquid-liquid extraction using a mixture of methanol (10 mL), pentane (50 mL) and PBS solution (1 mL). After phase separation, the upper layer (pentane) was re-extracted with 4 mL of PBS solution. Combined PBS and methanol extracts were further mixed with pentane (50 mL) and PBS solution (35 mL) for the second liquid-liquid extraction. With clear phase separation, the lower layer was filtered and an aliquot (40 mL) of the filtrate (equivalent to 4 g test portion) was mixed with 4 mL of 0.1 M citric acid and 36 mL of water and processed using immunoaffinity columns prior to the LC-FLD analysis.

Bourais et al. [73] developed a two-phase clean-up system to analyze aflatoxins in barley and milk based on partitioning of aflatoxins between toluene and an in-house aqueous solvent, PBS-methanol (1:1, *v*/*v*). Briefly, the aflatoxins were extracted from samples with methanol or chloroform. The resulting extracts were evaporated and reconstituted in toluene, a hydrophobic solvent, followed by the partitioning of aflatoxins to the PBS/methanol phase, while interferences remained in the organic phase. The partitioned aflatoxins were directly determined by FLD. However, such an approach required exhaustive optimization of partitioning conditions (e.g., combinations of different solvents at various ratios, extraction time) for different matrices, a common drawback of liquid-liquid extraction [74].

To minimize solvent consumption of conventional liquid-liquid extraction and increase concentration factors, liquid-liquid microextraction or dispersive liquid-liquid microextraction (DLLME) [75] has been increasingly used for the determination of aflatoxins in dairy and oil products [76,77,78,79]. As a miniaturization of liquid-liquid extraction, DLLME allows extracting and preconcentrating target aflatoxins into a very small amount (microvolume) of the extraction phase. For example, Campone [80] used a DLLME procedure for aflatoxin M_1_ determination in whole, skimmed and powdered milk with LC-MS. Aflatoxin M_1_ was extracted and salted out by the addition of sodium chloride and acetonitrile. After centrifugation, the acetonitrile supernatant was used as the dispersive solvent and extractant (chloroform, 1.5 mL) and water (5 mL) were added sequentially to perform DLLME. Hand shaking and centrifugation were used to facilitate extraction and achieve phase separation. The developed method using DLLME for aflatoxin detection in milk gave a method LOQ of 0.6 ng/kg, which is sufficient to measure aflatoxin M_1_ below the EU regulations (25–50 ng/kg). To improve extraction efficiency and accurately control the volume of extraction solution, Sun et al. [81] used a drop-on-demand inkjet device to inject extraction solvent (10 μL, chloroform) as ultrafine droplets (20 μm diameter) at high frequency into wheat extracts. The extraction could be finished within 1 min and recoveries of the aflatoxins (spiked at 0.05–0.5 µg/L) ranged from 83% to 93% with RSDs < 5%. However, evaporation and solvent exchange had to be performed to remove chloroform prior to the LC-MS analysis, which is a disadvantage when coupling DLLME with LC-MS.

Another interesting derivative of the liquid-liquid extraction is centrifugal partition chromatography, in which both stationary and mobile phases are liquids and a strong centrifugal force is used to immobilize a solvent on a stacked and interconnected rotating-discs as the stationary phase. Just like conventional liquid chromatography, whereby solvent (the mobile phase) and sample partition into a stationary phase, different analytes are eluted according to their partition coefficients in the two solvents. In a recent study, Endre et al. [82] reported the separation and purification of aflatoxins using centrifugal partition chromatography. After testing different biphasic systems, a toluene–acetic acid–water (30:20:50, *v*/*v*/*v*/%) solvent system was selected to provide optimized purification of aflatoxin B_1_, B_2_, G_1_ and G_2_ from an *Aspergillus parasiticus* fermented material in a 250 mL centrifugal partition chromatography column. Mass spectrometry was then used to confirm the identity of the purified aflatoxins and their purity was determined to be greater than 96% using LC-UV. Unfortunately, there was no sufficient method performance data regarding centrifugal partition chromatography-based aflatoxin analysis for naturally contaminated samples.

While liquid-liquid extractions possess the advantage of a simple operation and ease of apparatus handling, the methodology is often a cumbersome process requiring a large amount of organic solvent, especially when processing multiple sample extractions. To optimize extraction conditions, one has to exhaustively test various combinations of different solvents [83,84]. Liquid-liquid extractions are also prone to error resulting in analyte dilution from handling large solvent volumes and reduced recovery from the formation of sample emulsions. Due to the use of more effective clean-up (solid phase extraction (SPE) or immunoaffinity columns) and detection (LC-FLD and LC-MS) technologies with better sensitivity and specificity, liquid-liquid extraction has been significantly simplified and mainly used as an extraction technique. In general, a one-step extraction is performed for aflatoxins, followed by SPE, immunoaffinity clean-up or direct injection of the extraction [85,86,87,88,89,90].

#### 2.2.2. Solid Phase Extraction

After SPE columns became commercially available, SPE has been widely used as a replacement for conventional liquid-liquid extraction [91,92]. SPE achieves clean-up via selective retention of analytes while limiting interferences in extracts due to the careful selection of an adsorbent that would have strong analyte affinity. Compared to liquid-liquid extraction, SPE is more sample- and solvent-efficient, selective and flexible due to a wide range of adsorbents available for selection. For aflatoxin analysis, the most commonly used SPE cartridges contain silica gel, Florisil, C18 and other phases.

For the aflatoxin analysis, the early SPE applications were mainly focused on silica gel- and Florisil-based clean-up. Without commercially available SPE cartridges, researchers had to prepare in-house glass columns using activated silica gel [93], silicic acid [94], silica gel [95] or Florisil [96,97] as the adsorbent and different combinations of alcohols (methanol or ethanol) and chloroform as elution solutions. SPE conditions had to be determined by trial and error, varying by SPE columns, adsorbents (type and amount) and matrices. These early SPE procedures offered unsatisfactory separation between aflatoxins and matrix components and consumed a large amount of organic solvents due to inconsistent quality of adsorbents and packing. Very often, two SPE columns had to be used consecutively to purify aflatoxins. Robertson et al. [95] reported a stepwise procedure for the purification of aflatoxins from inoculated wheat. After the initial extraction and partitioning, the resulting crude mixtures of aflatoxins in chloroform (10 mL) was loaded on a glass column packed with silica gel (70 g, particle size, 0.05–0.20 mm) and capped with 25 g of anhydrous sodium sulfate. The column was washed with ethyl ether (500–600 mL) to remove pigments and other impurities, followed by eluting aflatoxins using 3% methanol in chloroform (600 mL). The collection of fractions was guided by intermittent inspection of the glass column with an ultraviolet lamp. The combined fractions were evaporated to dryness, but the resulting aflatoxins were further purified using a second column due to low purity (60%). In the second column, a slurry of silica gel and 20% methanol in benzene was used as an adsorbent permitting selective partitioning of aflatoxins on the silica gel. Aflatoxins were eluted in 1.75% methanol in benzene. The four aflatoxins (B_1_, B_2_, G_1_ and G_2_) were collected in different fractions and checked with TLC. In total, more than 100 fractions (20 mL each) were collected and 2 L of the eluting solvent was used.

To address insufficient sensitivity and selectivity issues of early SPE applications [91,98], Sobolev and Dorner [99] developed an SPE clean-up procedure using basic aluminum oxide as the adsorbent. Samples were extracted with methanol-water (80:20, *v*/*v*). An aliquot of the extract was mixed with acetonitrile (1:1, *v*/*v*) and pipetted onto an Alltech Extract-Clean cartridge packed with basic aluminum oxide where aflatoxins were eluted by gravity. The purified extract was injected onto an LC system with fluorescence detection. Recoveries of aflatoxins B_1_, B_2_, G_1_ and G_2_ from peanuts spiked at 5.0–2.5 mg/kg were between 80 and 87% with RSDs < 5%. The basic aluminum oxide sorbent retained compounds of medium to high polarity in polar solvents like methanol, acetonitrile and water, while significantly removing low polarity and nonpolar (both neutral and acidic) interferences from the extract. Since food matrices that are prone to aflatoxin contamination contain similar components such as water, carbohydrates, lipids and proteins, a careful selection of SPE sorbent chemistry is required to promote the purification of aflatoxins from co-extracted food matrix components prior to downstream analysis. Wilson and Romer [100] developed the Mycosep multifunctional clean-up column to retain co-extracted compounds that could create interferences in aflatoxin analysis but allow targeted aflatoxins to pass through. Test samples were extracted with acetonitrile–water (9:1, *v*/*v*) and resulting extracts were passed through the column. Aflatoxins were analyzed by LC-FLD. The column was tested using extracts from a variety of representative commodities such as corn, tree nuts, cereal grains, seeds and mixed feeds. The average recoveries of total aflatoxins were greater than 95% total aflatoxins and coefficients of variation were less than 3%, providing a selective one-step clean-up strategy for sample preparation.

Successful developments of SPE applications using different adsorbents are also reported in other studies. Ventura et al. [101] developed an SPE procedure for aflatoxin analysis in medicinal herbs. Samples were extracted with methanol-water (8:2, *v*/*v*) and passed through an Oasis HLB cartridge (packed with polymeric adsorbents) conditioned with methanol and equilibrated with water. After washing the cartridge with methanol-water (3:7, *v*/*v*), aflatoxins were eluted with methanol. The eluted extract was analyzed by LC-MS. Park and Kim [102] used Sep-Pak silica SPE tubes to clean up rice samples for aflatoxin B_1_ analysis. Rice samples were extracted using 80% methanol and the purified extracts were analyzed using LC-FLD. Recoveries were between 80 and 90% with RSDs < 6%. Using SPE packed with Florisil, Sobolev [103] purified aflatoxins from corn, brown and white rice, cottonseed, almonds, Brazil nuts, pistachios, walnuts and hazelnuts. Samples were extracted using methanol-water (8:2, *v*/*v*), followed by an SEP clean-up step. Eluted aflatoxins were quantified by LC-FLD. Recoveries of aflatoxins B_1_, B_2_, G_1_ and G_2_ were between 90 and 100% with RSDs < 5% in tested matrices. Romero-González et al. [104] developed a multimycotoxin method, in which aflatoxins B_1_, B_2_, G_1_, G_2_ and M_1_, fumonisin B_1_ and B_2_, deoxynivalenol, ochratoxin A, HT-2 and T-2 toxins and zearalenone were extracted from beer using C18 SPE cartridges. Key parameters such as the type of adsorbent, elution solvents and dilution factors were evaluated. After sonication, beer samples were directly loaded onto a C18 cartridge conditioned with a mixture of acetonitrile–methanol (6:4, *v*/*v*) and water. Mycotoxins were eluted by the same mixture of acetonitrile–methanol and analyzed by LC-MS. Recoveries of all targeted mycotoxins ranged from 76 to 106% with RSDs < 21%.

To simplify SPE, researchers have developed matrix solid-phase dispersion (MSPD) clean-up, a technique that does not need cartridges but directly mixes samples and adsorbents together. The mixture is then packed in a glass column, followed by the elution of target analytes [105]. The approach is generally applied to small test portions (e.g., 0.5–2 g) so it is better suited for instruments with sufficient sensitivity, otherwise additional evaporation and concentration steps are needed. Blesaa et al. [106] developed an MSPD procedure for the analysis of aflatoxin B_1_, B_2_, G_1_ and G_2_ in peanuts using C18 bonded silica as the dispersive adsorbents and acetonitrile as the eluting solvent. Eluted aflatoxins were concentrated via evaporation and quantified using LC-FLD and identity of aflatoxins were further confirmed using LC-MS. Recoveries of spiked aflatoxins at 2.5 and 5.0 ng/g ranged between 78 and 86% with RSDs < 10%. The LOQs were 0.125–2.5 ng/g.

Unlike conventional SPE, magnetic solid-phase extraction (MSPE) use superparamagnetic nanoparticles (e.g., Fe_3_O_4_) as an adsorbent. In MSPE, the adsorbent is directly added into the sample solution to extract analytes. After extraction, the adsorbent is separated using magnetic decantation via an external magnet. Therefore, no conditioning, washing or elution is needed. McCullum et al. [107] used polydopamine-coated magnetic nanoparticles (PD-MNPs) as the adsorbent to extract aflatoxins from wine. The nanoparticles were prepared using an in situ oxidative self-polymerization procedure and characterized using transmission electron microscopy (TEM) and Fourier-transform infrared spectroscopy (FTIR). Samples were first mixed with the PD-MNPs and then placed on a magnet to precipitate PD-MNPs. Absorbed aflatoxins were eluted from the PD-MNPs with acetonitrile–methanol (1:1, *v*/*v*). Under the optimized MSPE conditions, recoveries of aflatoxins ranged from 59% for aflatoxin G_2_ to 89% for aflatoxin B_1_. Best suited for extracting aflatoxins from liquid matrices, MSPE applications have additionally been investigated for the determination of aflatoxin M_1_ in milk [108].

For solid food matrices, samples need to be extracted, filtered and mixed with buffers, making the extracts compatible to the MSPE step because physicochemical properties of synthesized magnetic nanoparticles are sensitive to temperature, polarity and pH of the extracts. Hashemi et al. [109] applied MSPE for the determination of aflatoxins B_1_ and B_2_ in cereal products. In this study, magnetic nanoparticles coated with 3-(trimethoxysilyl)-1-propanthiol (TMSPT) and modified with 2-amino-5-mercapto-1,3,4-thiadiazole (AMT) were used as the adsorbent. Prior to the MSPE step, rice samples were extracted with a mixture of methanol: PBS solution (pH 7.4) and the resulting extracts were filtered prior to dilution with PBS solution. Corn samples were similarly extracted with an additional partitioning step using hexane. Such pretreatment steps, however, reduce the simplicity and efficiency of MSPE clean-up. Furthermore, magnetic nanoparticles with high selectivity and absorption efficiency are rarely commercially available. The cost, time and expertise required to synthesize and characterize magnetic nanoparticles contribute to the low attention to MSPE among mycotoxin labs.

The application of solid phase micro extraction (SPME) for aflatoxin analysis is another area of development worth noting. Originally, SPME was mainly coupled with gas chromatography to analyze volatile compounds but has been extended to LC analysis for semi- and non-volatile compounds [110,111]. Nonaka et al. [112] developed an online SPME procedure by placing an in-house SPME device (a porous capillary column with divinylbenzene polymer as the stationary phase, film thickness of 17 μm) between the injection loop and needle of the autosampler promoting selective elution of aflatoxins from sample extracts for LC-MS analysis. The SPME procedure showed a significant increase in sensitivity compared to the direct injection of the same sample extract. The within-day and between-day precision of aflatoxins at 1.0 ng/mL were below 3.3% and 7.7%. However, the whole process was highly customized and needed to use in-house software and hardware to operate the SPME device. In another study, Quinto et al. [113] used commercially available SPME devices coupled with LC-FLD for the determination of aflatoxins in cereal flours. Flour extracts were prepared using a methanol:phosphate buffer, followed by a SPME extraction using a fiber coated with polydimethylsiloxane/divinylbenzene (PDMS/DVB). Key parameters such as fiber polarity, temperature, pH, ionic strength, adsorption and desorption time, mobile phase, were evaluated. Under optimized conditions, the recoveries of aflatoxin B_1_ at 10 and 1 ng/g were 60% ± 7% and 67% ± 7% (14 h of extraction) and 52% ± 4% and 56% ± 2% (30 min of extraction), respectively, suggesting limited adsorption capacity of the fiber and the need of using an internal standard to compensate insufficient extraction efficiency. Currently, SPME is not widely used for aflatoxin analysis, though it has the potential to be automated. To make SPME more practical and applicable for aflatoxin analysis, one should address the high cost and limited selection of stationary phases of SPME in future studies.

#### 2.2.3. Immunoaffinity Column Clean-Up

From a burgeoning research area that focused on the discovery and isolation of monoclonal antibodies that could specifically recognize aflatoxins in the 1980s [114,115,116] to a well-accepted sample preparation practice in the 1990s [92,117,118], it took less than 10 years for antibody-based immunoaffinity columns (IAC) to become one of the most important and widely used clean-up tools for aflatoxin analysis. Immunoaffinity columns achieve clean-up based on the specificity of binding between antibodies and aflatoxins. The high affinity and specificity of antibodies for aflatoxins have been the cornerstone in the development of the various immunoaffinity columns. Owing to advances in the production of monoclonal antibodies with specificity and affinity, immobilization of antibodies on supporting beads and well-established sample preparation protocols, IACs are applicable to a wide array of food matrices for aflatoxin analysis [117]. Extensive research proves that IACs demonstrate a high degree of specificity in binding aflatoxins even in the presence of complex food and feed matrices [119,120], providing a methodology for simplifying sample analysis, especially when using non-selective detectors such as FLD [121,122].

The development of antibody-based immunoaffinity columns comprises the production of the antibodies, immobilization of the antibodies on inert supporting beads such as agarose gel or silica and packing of the beads in cartridges filled with phosphate buffer to maintain physicochemical properties of the antibodies. The evaluation of immunoaffinity columns focus on, but not are limited to, aflatoxin binding capacity and specificity, shelf life, solvent tolerance, cross-reactivity with matrix interferences and reversibility [123,124,125]. For sample clean-up, the initial sample extract is loaded on the immunoaffinity column. As the extract passes through the column, aflatoxins are retained on the column by the antibody, followed by a wash step to remove impurities due to nonspecific binding and the elution of aflatoxins using a solvent that disrupts the binding between the antibody and the aflatoxins.

Groopman and Donahue [126,127] reported the development and evaluation of an early immunoaffinity column for the aflatoxin analysis. After isolating an immunoglobulin M (IgM) monoclonal antibody that recognized aflatoxins B_1_, B_2_, M_1_ and the major aflatoxin-DNA adducts, they developed an immunoaffinity column by immobilizing the antibody on cyanogen-bromide-activated Sepharose 4B for the analysis of aflatoxins in biological and food samples. Their results clearly demonstrated the potential of monoclonal antibodies as an efficient and simple sample preparation tools for the purification of aflatoxins from food matrices.

With commercial immunoaffinity columns, researchers have amassed an enormous amount of knowledge on immunoaffinity chromatography and associated applications for the aflatoxin analysis. Immunoaffinity column clean-up offers the extraction of aflatoxins from various food matrices with simple aqueous mixtures (e.g., methanol/water), making the final extracts compatible with TLC [128,129,130], fluorometry, LC-FLD [121,131] and LC-MS for both single- and multimycotoxin determination without additional sample treatment.

In a collaborative study [121], immunoaffinity column clean-up coupled with solution fluorometry with bromine and an FLD was used to determine total and individual aflatoxins in corn, peanuts and peanut butter. Fourteen participating labs extracted samples using methanol-water (7:3, *v*/*v*) and diluted the resulting extracts to less than 30% methanol with water, followed by an immunoaffinity column clean-up step. At the three spiking concentrations, 10, 20 and 30 ng/g, recoveries of total aflatoxins were 81%, 81% and 83%, respectively with the RSD_r_ ranging from 5.2 to 17%, and the RSD_R_ ranging from 4.7 to 51%. Due to the satisfactory performance of the method, a well-known AOAC Official Method 991.31 [132] was published based on this multilaboratory validated immunoaffinity column clean-up and LC-FLD procedure.

Although immunoaffinity column clean-up is often coupled with LC-FLD, Stroka et al. [129] used immunoaffinity columns to clean up samples for TLC analysis. Samples were extracted using a mixture of methanol-water (8:2, *v*/*v*). The extracts were filtered and diluted using PBS buffer prior to loading on the immunoaffinity column. The IAC was washed sequentially using aqueous methanol (11%) containing 0.5% (Tween-20^®^) and water, followed by the elution of aflatoxins using methanol. Sample eluants (in methanol) were evaporated and reconstituted in a hexane–acetone–methanol solution for TLC analysis. This immunoaffinity column clean-up procedure successfully addressed the insufficient elimination of matrix interferences associated with some classical clean-up procedures such as SPE used for TLC [133,134].

Due to the co-occurrence of aflatoxins and ochratoxin A in some food (e.g., dried fruit, spices and cocoa, nutmeg and botanical roots), it was a logical extension from single class mycotoxin immunoaffinity columns to multimycotoxin immunoaffinity columns so that both aflatoxins and ochratoxin A could be purified on the same column, an approach that is more efficient than using two individual columns. Chan et al. [135] used a commercial immunoaffinity column (AflaOchra^TM^ HPLC immunoaffinity column) to develop a procedure that can simultaneously purify aflatoxins and ochratoxins from maize cereals, whole corn, maize snacks, corn flour, polenta and peanut butter. Average recoveries (within-batch) were 72–94% for ochratoxin A and 73–101% for aflatoxins spiked at 5 µg/kg. RSD_r_ and RSD_R_ of aflatoxins and ochratoxin A in maize cereals were 7.6–10.1% and 10.2–13.8%, respectively. In this work, the estimated method detection limits of aflatoxins and ochratoxin A were around 0.2 µg/kg. Trucksess et al. [131] compared the method performance using a commercial multimycotoxin column (for both aflatoxins and ochratoxin A) and one single-mycotoxin column (for aflatoxins only). The two types of columns were operated in a very similar manner, and aflatoxins and ochratoxin A were measured using same LC-FLD. Results for aflatoxins (2–16 ng/g) using the single mycotoxin IAC clean-up were comparable (80% recovery) for ginseng and other botanicals such as ginger, liquorice and kava-kava. Using IAC clean-up for both aflatoxins and ochratoxin A, recoveries of aflatoxins in ginseng were about 70% but around 60% in ginger, liquorice and kava-kava. However, recoveries of spiked ochratoxin A (4 ng/g) in these botanicals were about 55%. Based on the results, another single-laboratory study [136] and a multilaboratory collaborative study were conducted to evaluate the performance of the multimycotoxin immunoaffinity column [137]. Similar applications of the multimycotoxin column have been extended to other food matrices such as peanut butter, maize, polenta, maize snacks, corn flour [135], bee pollen [138], olive oil [139] and cocoa product [140].

In recent years, multimycotoxin immunoaffinity columns have been developed and coupled with LC-MS to simultaneously screen for aflatoxins, deoxynivalenol, ochratoxin A, fumonisins, HT-2 and T-2 toxin and zearalenone in one sample clean-up step and instrumental analysis. Maria et al. [141], Vaclavikova et al. [142], Wilcox et al. [143] and Ye et al. [144] demonstrated successful applications of commercial multimycotoxin immunoaffinity columns in various foods. Although the immunoaffinity columns could achieve simultaneous clean-up of multiple mycotoxins, it has been a challenge to achieve satisfactory extraction efficiency of all targeted mycotoxins with different physicochemical properties in a single extraction, where the use of multiple extractions would defeat the purpose of a multimycotoxin analysis. Another practical issue with multimycotoxin immunoaffinity columns is the capacity of mycotoxins purification. Clean-up of more mycotoxins on one column means less flexibility and more stringent column conditions without comprising of clean-up efficiency for multiple mycotoxins. The complexity and cost of column production increases with the number of different types of antibodies that need to be immobilized. Furthermore, it was expected that immunoaffinity columns could provide a clean extract with marginal matrix effects on LC-MS. Unfortunately, that is not the case and making matrix-match calibration standards using immunoaffinity columns is not a practical or economical practice. Therefore, in the future, study efforts may be dedicated to address these challenges to better utilize immunoaffinity column clean-up and LC-MS for aflatoxin analysis.

A noteworthy attempt associated with immunoaffinity columns is to automate the clean-up procedure using robotic devices. Sharman and Gilbert [145,146], Urano et al. [147] and Carman et al. [148] developed and evaluated in-house automated immunoaffinity column clean-up devices. In terms of clean-up efficiency and sample throughput, their results were comparable to manual procedures. These early devices could replace human operators to perform sample clean-up to some extent, but the degree of automation and robustness was low. These devices lacked the capability of accurately dispensing liquid extracts or troubleshooting common errors as human operators. Sample extractions still needed to be performed manually and transferred to the device. Software and hardware were not user-friendly, making training, daily operation and maintenance a burden for human operators. So far, automated sample preparation has been far from a popular option due to the complex and challenging nature of aflatoxin analysis in foods. Hopefully, researchers will continue to take advantage of the advances in computing and engineering technologies and develop automated clean-up procedures for aflatoxin analysis in the near future [41,149].

#### 2.2.4. Supercritical Fluid Extraction (SFE)

Carbon dioxide (CO_2_) has a low critical temperature (31 °C) and pressure (7.3 MPa) [150], which makes CO_2_-based supercritical fluid extraction (SFE) an ideal tool for extracting thermally liable, non-polar analytes from foods [151]. Since the 1990s, SFE attracted some attention as an alternative extraction technique for the aflatoxin analysis [152,153,154,155,156,157,158]. Compared to conventional liquid extraction techniques or other energy-assisted extraction techniques, SFE offers unique advantages: it minimizes the use of organic solvents, offers easy separation of extraction solvent (CO_2_) and extractants (aflatoxins) by simply releasing the pressure and has potential to be connected with analytical instruments, forming online analysis and eliminating manual sample handling and transfer steps [159].

Arguably, as a group of relatively polar compounds, aflatoxins are not perfect analytes for SFE so it was no easy task to develop SFE protocols for the aflatoxin analysis. Researchers had to increase SFE pressure and temperature and add modifiers to increase solubility of aflatoxins in CO_2_ but these manipulations also contributed to an increase in co-extracted non-polar and fatty interferences, requiring additional clean-up procedures after SFE. According to a study by Taylor et al. [153], the optimized SFE conditions, 5000 psi, 80 °C, 15% modifier (acetonitrile–methanol, 2:1) and 100 mL of liquid CO_2_, gave excellent recoveries (94.6% with RSD 6.2%) for aflatoxins extracted from field-inoculated corn, but extensive clean-up was needed to remove co-extracted interferences in the SFE extracts. Similar issues have been reported in other matrices with relative high fat content such as peanut, liver and animal feeds [152,154,156]. It is worth noting that when applying SFE to low-fat matrices, less clean-up is needed. Liau et al. [158] used SFE to extract aflatoxins from *Zizyphi fructus*, a traditional Chinese medicine and the resulting extracts were directly injected into LC-MS. Although SFE has been around for many years, the development of new SFE-based aflatoxins methods has been relatively slow. The expensive capital instruments, complex operation/training and time-consuming method development all contribute to the poor uptake of SFE in sample preparation for the aflatoxin analysis.

#### 2.2.5. Energy-Assisted Extraction

Energy-assisted liquid extraction techniques, such as Soxhlet extraction, accelerated solvent extraction and microwave-assisted extraction facilitate sample extraction by applying energy (heat and microwave). As a mass transfer of aflatoxins between the sample and extraction solvent increases with applied energy, temperature and/or pressure, these extraction techniques can provide excellent extraction efficiency, significantly reducing extraction time and consumption of extraction solvents. Energy-assisted extraction for aflatoxin analysis started with Soxhlet extraction, a semiautomated extraction process that could circulate the extraction solvent to repetitively extract the sample using specialized glassware. The exhaustive extraction durations were cumbersome and co-extracted matrix components need to be removed afterwards [160,161].

Microwave-assisted extraction and accelerated solvent extraction [162] have also been evaluated for aflatoxin analysis. Unlike Soxhlet, these energy-assisted extraction techniques facilitate aflatoxin transfer from samples to extraction solvents by applying high temperature and pressure in a more controlled and efficient manner, improving extractability and accessibility of aflatoxins with a much shorter extraction time. However, using these technologies additional matrix components are prone to co-extraction and additional steps are required to clean-up the resulting extracts due to the non-selective and aggressive nature of these extraction techniques. It is not surprising that only a handful of applications displaying microwave-assisted extraction and accelerated solvent extraction have been developed for aflatoxin analysis [163,164,165,166]. Compared to conventional liquid extraction, these technologies demonstrate limited advantages other than efficiency. The cost and maintenance of the instruments for accelerated solvent extractions and microwave-assisted extractions are significantly higher than conventional lab glassware used for liquid extraction. Unless better selectivity or online sample clean-up features could be incorporated with these energy-assisted extraction devices, they would remain as research-oriented devices but adopted for the routine aflatoxin analysis.

## 3. Chromatography

### 3.1. Thin Layer Chromatography

As one of the early chromatographic techniques used for aflatoxin analysis, TLC was credited for its important role in the first purification and identification of aflatoxins back in the 1960s [94,167] and since then various TLC applications have been developed, in a large part due to its simple and economical nature [168].

As a simple but inefficient chromatographic technique coupled with non-specific detection [169], early TLC methods were vulnerable to fluorescence interferences and non-fluorescing materials of similar migration on the plate (R_f_ value) that could mask aflatoxins [170,171]. Numerous studies were conducted to improve separation capacity of TLC. Noteworthy developments in TLC are high-performance TLC (HPTLC), 2-dimentional TLC (2D-TLC) and over-pressured layer chromatography (OPTLC) [172,173]. As an extension of conventional TLC, HPTLC increases the resolution of aflatoxins by using high quality plates coated with stationary phase with uniform and fine particles. With automated sample application devices and densitometers for quantitative analysis, HPTLC provides more efficient separation and better sensitivity [174]. Using HPTLC and multiple developments with two solvent systems of different polarity, Lee et al. [175] achieved base-line separation of aflatoxin B_1_, B_2_, G_1_, G_2_, M_1_ and M_2_ and the other seven mycotoxins within 1 hr. Detection limits of aflatoxins were in the low picogram ranges by fluorescence. Quantitation in the nanogram range demonstrated good precision with RSDs ranging from 0.7 to 2.2%.

In 2D-TLC analyses, the plate is turned 180° after the first development and aflatoxins and matrix components are further separated in the 2nd development. The comparison of 1D-TLC and 2D-TLC for aflatoxin M_1_ and B_1_ in fatty foods (milk and eggs) shows that the two techniques provide comparable recoveries, but RSDs associated with 2D-TLC are much lower (7%) than that of 1D-TLC (16%), suggesting better repeatability [176]. The author emphasized that with limited separation capacity, 2D-TLC-based determinations of aflatoxin M_1_ and B_1_ requires in-depth sample clean-up procedures for fatty samples but the TLC analysis itself is an easy operation for inexperienced chemists and a more economical choice for labs without a budget for capital instruments such as LC. Many AOAC official methods were developed based on 2D-TLC for aflatoxin analysis in complex matrices [169].

Invented in the 1970s [177], OPTLC has been commonly described as a planar layer version of HPLC, in which the mobile phase is pumped through a thin column with a much wider cross-section than conventional LC columns [173]. The applications of OPTLC has been limited to research applications, most likely due to the complexity of the system. In order to perform OPTLC, one would need a pressurized chamber, a liquid-delivery system, a detector in addition to other ancillary components. Although, advanced features such as online sample cleanup and delivery could be incorporated in to OPTLC, over-engineering would eliminate the simplicity of TLC with underperforming separation efficiency and sensitivity compared to LC. To date, only a handful of OPTLC applications are reported for aflatoxin analysis [172,178,179].

Generally, in terms of identification, a comparison of migration distance ratio, R_f_ values (samples vs. standards) on the TLC plate can only provide presumptive evidence of the existence of aflatoxins, and further confirmation tests are required, especially for compliance samples. Andrellos and Reid [180] developed a confirmation procedure based on the fluorescent reaction products of aflatoxin B_1_ with formic acid-thionyl chloride, acetic acid-thionyl chloride and trifluoroacetic acid. The procedure required a large on plate mass of aflatoxin B_1_, yet it did not work for other aflatoxins. Attempts to couple TLC with mass spectrometry for more specific identification were reported by Haddon et al. [181,182], Brumley et al. [183], Park et al. [184] and Tripathi et al. [185]. However, aflatoxins were unable to be directly analyzed by MS requiring extensive clean-up steps to remove aflatoxins from the plate and purified prior to MS analysis. These procedures were time consuming and instrument setups were cumbersome and expensive.

In terms of quantitation, TLC visual estimation cannot generate accurate quantitative results. A collaborative study also reported large variability among participating labs using TLC [186]. In some cases, the measured errors could go up to 56% and precisions ranged from 20 to 28% [187,188]. The development of fluorodensitometric measurements of TLC spots can improve the quantitative analysis of aflatoxins in terms of precision and accuracy [189], however, such experiments sacrifice throughput because only one spot can be scanned at a time. One of the advantages of TLC is that multiple samples could be analyzed in a parallel manner, while even modern LC-MS has to run samples sequentially. Other issues related to TLC such as variability in sample spotting, silica gel adsorption activity and silica gel particle size also lead to unreliable results. Furthermore, temperature and humidity in the lab could affect TLC performance and aflatoxins could be photochemically degraded under excess UV lights [190,191]. It is not surprising that these inherent disadvantages of TLC gradually contributed to the replacement of TLC with LC-FLD and other advanced techniques.

### 3.2. Liquid Chromatography

In the 1970s, there were increasing interests in LC-based aflatoxin analysis. It was expected that LC had the potential to achieve faster separations with better accuracy and precision than TLC. To realize the potential, considerable efforts were invested to evaluate column and detector (UV and FLD) performance. Aflatoxin B_1_ and B_2_ exhibit strong blue fluorescence (425 nm) and aflatoxin G_1_ and G_2_, green fluorescence (450 nm). Fluorescence spectroscopy was preferred to UV-Vis absorption, due to its higher specificity and improved detection [192]. To take advantage of this physicochemical property, the majority of researchers couple LC with fluorescence detectors for the identification and quantitation of aflatoxins.

Initially, normal phase LC was used for aflatoxin analysis. Commercial pumps and columns were not well-developed yet, so separation efficiency was limited, resulting in overlapping chromatographic peaks for aflatoxins [193,194]. To improve resolution and shorten separation time, Garner [195] achieved the baseline separation of aflatoxin B_1_, B_2_, G_1_ and G_2_ on a silica gel column (25 cm × 3 mm I.D. × 6 μm) within 15 min. In this work, water-saturated dichloromethane with 0.3% *v*/*v* methanol was used as a mobile solvent. However, the method did not have sufficient sensitivity due to the use of UV detection, requiring an on-column mass of 10 ng for the detection of aflatoxins. Seitz [196] evaluated the separation of aflatoxin B_1_, B_2_, G_1_ and G_2_ on three different types of commercial columns, among which a prepacked silica gel column (6.35 mm O.D. × 30 cm, 10 μm silica gel) provided satisfactory separation. Chloroform–methylene chloride (75:25, *v*/*v*) containing 0.50% methanol was used as the mobile phase. Worth noting, the method was more sensitive for aflatoxin G_1_ and G_2_ than aflatoxin B_1_ and B_2_ because chloroform, dichloromethane and/or methanol solvent systems quenched fluorescence of aflatoxin B_1_ and B_2_, an issue that was resolved later by using different detectors, special mobile phases and a flow cell packed with silica-gel particles.

Pons and Franz [70] coupled UV (for the detection of aflatoxin B_1_ and B_2_) and FLD (for that of aflatoxin G_1_ and G_2_) with a normal phase LC so that the four aflatoxins could be simultaneously monitored. This allowed the detection of all four aflatoxins at levels below 1.0 ppb, although it was somewhat cumbersome to program two detectors. Manabe et al. [197] proposed using a mobile phase (toluene–ethyl acetate–formic acid–methanol, 89.0: 7.5: 2.0: 1.5 *v*/*v*/*v*/*v*), solvent system that did not quench the fluorescence of aflatoxin B_1_ and B_2_. The method could detect aflatoxins in foods as low as 10–20 ppb. Alternatively, Zimmerli [198] and Panalaks and Scott [199] used a flow cell of the fluorometric detector filled with silica-gel particles to extract aflatoxins from the mobile phase after LC separation. In this work, FLD detection had the same physical characteristics as that on a TLC plate. Aflatoxins were absorbed and detected on a silica-gel particles solvent, thereby minimizing quenching of fluorescence. Although the addition of the silica gel particles into the flow cell slightly impacted chromatography (e.g., peak broadening), the approach was commercialized and widely integrated with LC-FLD as an indispensable feature for aflatoxin analysis in foods [69,200,201,202].

In the 1980s, reversed phase LC rapidly replaced normal phase LC for aflatoxin analysis. LC separation of aflatoxins on reversed phase columns was straightforward. Generally, mixtures of water, methanol and/or acetonitrile were used as the mobile phase, promoting aflatoxin separation using isocratic conditions. Fluorescence quenching in mobile phases remained a challenge for the detection of aflatoxins at sub ppb levels in foods. Thee fluorescence emission of aflatoxin B_1_ and G_1_ could be significantly quenched by aqueous mobile phases utilized in reverse phase LC. To overcome this limitation, a common practice is to derivatize aflatoxins, minimizing the impact of signal quenching. Either precolumn derivatization with trifluoroacetic acid (TFA) or post-column derivatization with a halogen (bromine or iodine), are commonly employed, adding one hydroxyl group or two atoms of the halogen at the 8,9-double bond of the dihydrofuran moiety of aflatoxins. Alternatively, post-column photochemical derivatization can be used for aflatoxin analysis.

The findings that aflatoxin B_1_ can react with TFA, forming a fluorescing product, aflatoxin B_2_a in aqueous solvents [203,204] paved the way for TFA-based derivatization, which was mainly used prior to LC separation. Pons et al. [205] thoroughly investigated the hydration of aflatoxin B_1_ and G_1_ in acidic aqueous solutions in terms of pH, reaction times/rates and product stability. The study confirmed that the double bonds in the dihydrofuran moieties of the aflatoxins B_1_ and G_1_ could be hydrated, generating reaction products with sufficient fluorescence for detection. However, the study also demonstrated these products were not stable and gradually degraded into non-fluorescing compounds.

Takahashi [188] developed and optimized a TFA-based derivatization for the analysis of aflatoxins in wine. Aflatoxins were extracted from spiked wine using methylene chloride and the concentrated sample extracts were cleaned up on a silica gel-alumina column. After evaporation, the remaining extracts were reacted with TFA for a few seconds, followed by the addition of 10% acetonitrile and LC-FLD analysis. Derivatization products, aflatoxin B_2a_, G_2a_ and two native aflatoxins, aflatoxin B_2_ and G_2_ were separated on a 25 cm × 3.2 mm I.D., 10 μm ODS-bonded silica gel column within 15 min. A mixture of methanol and water was used as the mobile phase. Recoveries of the four aflatoxins spiked at 1 ppb ranged from 88%-106%. The method could detect aflatoxins in wine as low as 0.02 μg/L, 8× more sensitive than TLC [206]. The procedure has been extended and modified for different food matrices [207,208]. For example, using the same derivatization procedures, Beebe [209] developed an LC-FLD method for aflatoxins analysis in green coffee and peanut butter. Recoveries of aflatoxins spiked at 1–15 ppb ranged from 72 to 104%; Diebold and Zare [210] replaced TFA with hydrochloric acid for derivatization and achieved the detection of aflatoxins at subpicogram levels in corn using a laser fluorescence detector; Beebe and Takahashi [211] derivatized aflatoxin M_1_ extracts from milk and dairy products using TFA, followed by an LC-FLD analysis. Aflatoxin M_1_ could be detected at as low as 0.3 ppb and recoveries were 93–125% at 1 ppb in spiked matrices. An AOAC official method was also developed for aflatoxin analysis in corn and peanut butter using TFA precolumn derivatization, a C18 analytical column and water–methanol–acetonitrile as the mobile phase [212]. TFA derivatization was companied by considerable disadvantages. These included peak broadening caused by residual TFA and instability of the derivatization products, aflatoxin B_2_a and G_2_a in methanol [205,208,213]. Additionally, TFA derivatization had to be conducted offline prior to LC separation, making it less desirable compared to post-column derivatization, which could be integrated with LC-FLD as an online operation.

Similar to precolumn derivatization, post-column derivatization started with the finding that iodination of aflatoxin B_1_ and G_1_ exhibit strong fluorescence [214]. Tuinstra and Haasnoot [215] developed a prototype post-column derivatization device, in which an iodine solution was pumped from a reservoir and mixed with eluted aflatoxins in a reaction coil (3000 mm × 0.5 mm, 60 °C) inserted between the LC column and FLD. This led to a 50× increase in sensitivity of aflatoxins. Encouraged by the promising results, researchers conducted a series of studies to optimize this post-column derivatization method, focusing on compositions of the mobile phase, iodine reagent flowrates, reaction temperatures, iodine concentrations, confirmation of derivatization products using MS, etc. [216,217,218,219,220,221,222,223].

Despite the wide acceptance of iodine-based post column derivatization, Kok and his colleagues [224,225,226] developed another online post-column derivatization based on bromination of aflatoxins to simplify derivatization steps and lower instrument cost. In their approach, bromine is generated from bromide (e.g., potassium bromide) added into the mobile phase and reacted with eluted aflatoxins in a post-column electrochemical cell, namely KOBRA-cell, prior to FLD analysis. The bromine concentration can be carefully controlled by the generating current, maximizing fluorescence intensity. Using this approach, aflatoxins could be detected at an on-column mass at 20–40 pg.

Other valuable signal-enhancement techniques were also developed [227,228,229,230]. Garner et al. [227] developed a more cost effective bromination method using pyridinium bromide perbromide as the derivatization agent. To further increase sensitivity, Chiavaro et al. [228] and Maragos et al. [229] evaluated the addition of cyclodextrins as a fluorescence enhancer. Joshua [230] reported the first photochemical derivatization method of aflatoxins. Compared to chemical derivatization, this approach does not need the addition of any chemical reagents. Aflatoxins were derivatized using UV generated by a photochemical reactor and could be detected in corn below 1 ppb. In recent years, there have been notable efforts to evaluate and compare photochemical derivatization to chemical derivatization. Studies by Papadopoulou-Bouraoui et al. [231] and Waltking et al. [232] demonstrate photochemical derivatization is as efficient as chemical derivatization using bromine or iodine.

With the advent of commercial UHPLC, there are some applications of UHPLC-FLD for aflatoxin analysis. Due to the use of columns packed with submicron (<2.0 μm) particles, rapid and efficient separations could be achieved. The speed and high resolution of UHPLC made it an attractive tool for analyzing mycotoxins in foods [233,234]. Ibanez et al. [235] developed an UHPLC-FLD method to simultaneously determine aflatoxins, OTA and zearalenone in barley. Photochemical derivatization was performed to enhance signal intensity of aflatoxins without impacting ochratoxin A and zearalenone. Iodination or bromination, however, is not suited for multimycotoxin analyses as ochratoxin A and zearalenone are unable to survive the derivatization process. The author also mentioned that the impact of the void volume (0.25 mL) introduced by the photochemical coil on chromatography should be monitored, though it is not an issue for conventional columns with bigger particle sizes.

Addel-Azeem et al. [236] used UHPLC-FLD to screen aflatoxins in food samples collected from local markets. Without any pre- or post-column derivatization, the method could detect individual aflatoxins as low as 0.02 ppb. An isocratic program was used to separate aflatoxins on a C18 column (2.1 mm × 100 mm, 1.7 μm). Their results suggest there were significant retention time shits of aflatoxins in solvent and sample matrices. Interestingly, the method seems more sensitive for aflatoxin B_1_ and B_2_ than aflatoxin G_1_ and G_2_. Banerjee and his colleagues [41,237,238] extended UHPLC-FLD-based aflatoxin analysis to both food and feed matrices. Using a FLD flowcell (13 μL) and a large injection volume (10 μL), these methods achieve the detection of aflatoxins below 1 ppb even in feed matrices without performing conventional derivatization. Due to its sensitivity capabilities and ease of use, these newly developed LC-FLD methods continue to attract mycotoxin labs to adopt them for routine sample analysis.

### 3.3. Liquid Chromatography and Mass Spectrometry

Since the 1980s, LC-MS has become the fastest growing technique available for mycotoxin analysis. The potential benefits of LC-MS-based mycotoxin analysis have long been recognized and exploited. The technique can achieve simultaneous multimycotoxin determination based on LC separation and mass to charge (*m/z*) of target analytes, an intrinsic property that provides more specific identification based on molecular weight of the target analyte than ultraviolet and visible light absorption or fluorescence emission, extrinsic properties used by LC-UV/FLD. In the 1980s, the limitations that hindered the application of LC-MS were interfaces between LC and MS detectors, limited ionization efficiency of MS detectors and the poor and unstable response of many mycotoxins due to the incomplete desolvation. The advent of commercial LC-MS instruments equipped with electrospray ionization has brought the identification and quantitation of contaminants, mycotoxins and pesticides in foods to another level [239,240]. The impact of modern LC-MS has been signified by the unmatchable sensitivity in quantitation, specificity in identification and number of mycotoxins that could be analyzed in one analysis. Currently, these highly desirable features make LC-MS the gold-standard for mycotoxin analysis [241].

#### 3.3.1. LC-MS as Confirmatory Tool

Early efforts to use LC-MS for aflatoxin anaysis focused on thermospray MS coupled with LC [242,243]. As a soft ionization technique, thermospray MS could identify aflatoxins based on their molecular ions. Combined with retention times on the LC, the technique was believed to be a useful tool for confirmation. Hurst et al. [244] carefully characterized a commercial thermospray MS for the detection of aflatoxin B_1_, B_2_, G_1_ and G_2_ in peanuts. LC separation was achieved within 12 min on a C18 (4.6 mm ID × 25 cm, 5 µm particle size) column with 0.1 M ammonium acetate-methanol-acetonitrile (56:22:22, *v*/*v*/*v*) as the mobile phase and a flow rate of 1.0 mL/min. The identification and sensitivity of aflatoxins in sample extracts were evaluated using both full scan and selected ion monitoring (SIM) modes. Using the SIM mode, aflatoxins could be detected below 1 ng (on column mass). Although it was less sensitive than LC-FLD, the study demonstrated that LC-MS could, at least, be used as a confirmatory tool. In the meantime, using thermospray MS, Holcomb et al. [119] obtained the structural information and molecular weights of derivatized aflatoxins, aflatoxin B_1a_ and B_2a_, confirming the mechanism of the post-column chemical derivatization using iodine, which has been widely used to enhance fluorescence of aflatoxin B_1_ and G_1_ for the LC-FLD analysis. Another notable attempt was the hyphenation of a particle beam interface with electron impact (EI) ionization MS by Cappiello et al. [245]. Aflatoxins were introduced into the ion source via a reversed phase capillary column. The resulting full scan mass spectrum could be used for identification and improved sensitivity was achieved using the SIM mode for quantitation with estimated method detection limits around 10 ppb for individual aflatoxins.

Although these early LC-MS methods could not provide sufficient sensitivity for quantitative analysis of aflatoxins in foods, they were appreciated as a tool for confirmation with a high degree of specificity [246,247]. Back in the 1960s, researchers already realized the importance of confirming the identity of “presumptive” aflatoxins in positive samples. Extra steps of using another method to confirm the identity of detected aflatoxins were cumbersome but necessary when results are required to support sample compliance. Initially, aflatoxin confirmation was performed by bioassays [248], which was an indirect and time-consuming approach and soon replaced by techniques based on physicochemical properties of aflatoxins (e.g., 2D-TLC, chemical derivatization and FLD) [211,249,250]. When MS became available, negative chemical ionization MS- [182], gas chromatography (GC)–MS- [251] and eventually, LC-MS-based confirmation was adopted [136,252] by private and government laboratories.

#### 3.3.2. LC-MS for Quantitative Analysis

Since the later 1990s, modern LC-MS instruments, especially LC-triple quadrupole (LC-QQQ), has been developed with increasing sensitivity for quantitative analysis of various organic chemicals in foods. Despite high capital costs of LC-MS instruments, many efforts have been exerted to quantitate aflatoxins using this technique [253]. One of the challenges of LC-MS based mycotoxin analysis is matrix effects. In the course of the LC-MS analysis, signals of aflatoxins could be significantly suppressed by coeluting matrix components, an observation often referred to as “matrix-effects” [254]. If solvent-only calibration standards are used for quantitation, the differences in signal suppression or enhancement in solvent, which is free of co-eluted matrix components, compared to a sample matrix could lead to inaccurate quantitative results. Suppression is mainly caused by insufficient ionization of aflatoxins due to the competition of charges from co-eluted matrix components in the ion source. Matrix effects can be experimentally circumvented by diluting the sample matrix, in-depth cleanup, standard addition, matrix-matched calibration and/or the use of internal standards, especially ^13^C-labeled aflatoxins [255], but the solution is a balance of data quality, sample throughput and cost [256].

Dilution seems to be a convenient approach to mitigate matrix effects, but it requires sensitive LC-MS [257] and dilution factors are matrix-, instrument- and analyte-dependent. Additionally, regulatory levels of aflatoxins should not be overlooked. For example, the current FDA action level of aflatoxin M_1_ is 0.5 ppb in milk. If a 10× dilution is necessary to eliminate matrix effects, the LC-MS instrument must be able to quantitate the aflatoxin below 0.05 ppb [89]. When factoring in EU’s regulatory levels of aflatoxin M_1_ (0.025–0.05 ppb), more sensitivity is required if samples are diluted prior to LC-MS analysis. While such sensitivity is not possible for the majority of commercial LC-MS instruments, concentration and clean-up steps are necessary to ensure the detection of aflatoxin M_1_ at and below the regulatory levels [258,259]. In-depth sample clean-up could be used to eliminate matrix-components/interferences but very often the process is lengthy and laborious. Sometime, even with immunoaffinity columns, LC-MS analysis was not free from matrix effects [142]. Matrix-matched calibration standards are commonly used to achieve satisfactory quantitative results. In this approach, known amounts of aflatoxins are spiked into “clean” matrix and used as calibration standards. This is expected to bring same/similar impact of matrix effects on the response of aflatoxins in both calibration standards and samples. The preparation of matrix-matched calibration standards is tedious, especially when one needs to deal with a wide variety of food matrices [57,260]. Standard addition would be straightforward only if one analyte needs to be quantitated. When using the approach to quantitate the four aflatoxins at various concentrations in a sample, it becomes a challenging operation for analysts. The ideal solution for matrix effects would be stable isotope dilution assay (SIDA). Using commercially available ^13^C-aflatoxins or deuterated aflatoxins as internal standards (IS), one could quantitate native aflatoxins with solvent only calibration standards [256]. Very often extraction solvents and cleanup procedures have to be optimized depending on the matrix to ensure extraction efficiency. The incorporation of labeled IS into the sample prior to extraction and clean-up provides greater flexibility in sample extraction conditions across multiple sample matrices.

Vahl and Jørgensen [261] developed an LC-MS method for the determination of aflatoxins B_1_, B_2_, G_1_ and G_2_ in food and interestingly, they used aflatoxin M_1_ as the internal standard. Samples were extracted with methanol and further cleaned by column chromatography, previously developed for LC-LFD. On the LC-MS, recoveries for the aflatoxins spiked at 2 ppb in figs, peanut butter, peanuts, chili, pepper and curry ranged between 40 and 270%. Variability of individual aflatoxins was also unusually high. This study is a reminder of the challenges associated with LC-MS, especially matrix effects, demonstrating how clean-up procedures that work well for LC-FLD might not work for LC-MS. Cavaliere et al. [262] used aflatoxin M_1_ as IS for the determination of aflatoxins in corn. Samples were extracted using acetonitrile–water (80:20, *v*/*v*), followed by dilution and SPE cleanup. Aflatoxins were detected by LC-MS/MS. Quantitation was achieved by matrix-matched standards. Recoveries ranged from 81 to 101% with RSDs < 12% and the method LODs were from 0.1 to 0.6 ppb. Using internal standards is a good practice, but whether the selected internal standard would behave similarly to target analytes in different matrices should be carefully evaluated. Without an appropriate internal standard or matrix-matched calibration standard, one would not be able to effectively minimize the impact of matrix effects on quantitation.

Cervino et al. [263] synthesized and characterized deuterated aflatoxins B_2_ and G_2_ and used them to compensate the loss of analytes in the course of extraction and matrix effects for LC-MS-based aflatoxin analysis. The two deuterated IS were spiked into samples (almonds and wheat flour) prior to extraction so excellent recoveries and precisions were achieved. Recoveries were between 90% and 105% for all the four aflatoxins with coefficients of variation (CV) of 3.6%, 4.8%, 12% and 14% for aflatoxin B_1_, B_2_, G_1_ and G_2_, respectively. As expected, labeled B_2_ and G_2_ were more suited for the quantitation of aflatoxin B_2_ and G_2_ than B_1_ and G_1_ due to similar physicochemical properties between the labeled IS and the native aflatoxins (B_2_ and G_2_) and retention times on LC.

Li et al. [264] developed an isotope dilution LC-MS method for the determination of aflatoxins in feeds. Samples were extracted using methanol-water (70:30, *v*/*v*) or acetonitrile–water (84:16, *v*/*v*) depending on feed matrices, followed by filtration and dilution. Prior to LC-MS analysis, the extracts were spiked with ^13^C-aflatoxin B_1_ and aflatoxins were separated on a C18 (2.1 mm × 50 mm, 1.7 µm) column. Detection was performed using a triple quadruple MS. Identification of aflatoxins was achieved by comparing the retention times and relative ion ratios of the two MRM transitions in standards and samples [265]. Quantitation was based on linear least squares calibration of the relative response ratio of an aflatoxin and ^13^C-aflatoxin B_1_ plotted versus concentration. Recoveries of aflatoxins range from 78 to 122% with intra-day and inter-day precisions of 2–15% and 3–17%, respectively. In this study due to the concern of cost, only one ^13^C-aflatoxin was used and spiked into the sample extracts right before LC-MS analysis. Had ^13^C-IS been spiked into the samples prior to extraction, probably only one extraction solvent would have been used. As to the cost of ^13^C-IS, people often overlook the savings in labor cost and increase in sample throughput [255].

To take advantage of the sensitivity of LC-MS, Xavier and Scussel [266] used a “dilute-and-shoot” procedure to prepare samples for aflatoxin analysis in Brazil nut. Homogenized samples (25 g) were first extracted using 100 mL of acetonitrile–water (80:20, *v*/*v*) and further diluted 4× using water prior to LC-MS analysis. Matrix-matched calibration standards were used for quantitation. Recoveries of spiked aflatoxins at 1–10 ng/g ranged between 92 and 100% with RSDs < 10%. The method combined a simple sample preparation procedure and an effective solution to matrix effects together in a practical manner. Similar approaches have been reported in other studies [267,268]. Breidbach and Ulberth [269] developed a highly accurate quantitation method for aflatoxin B_1_ using 2D-LC and double isotope dilution MS. Aflatoxin B_1_ was carefully separated from matrix-components using heart-cutting 2D-LC and measurement uncertainty related to extraction efficiency and matrix effects was minimized using ^13^C-IS. In tested matrices (cereal-based baby food, maize and maize-based feed), the expanded uncertainty of aflatoxin B_1_ at 0.197 ppb is 0.017 μg/kg (8.9%). The application of 2D-LC-MS might not be suited for routine sample analysis but is appreciated for measurements that require high accuracy such as characterization of certified reference materials. Recently, Campone et al. [270] reported determination of aflatoxins, ochratoxin A, fumonisins B_1_ and B_2_ in beer using 2D-LC-MS. For fumonisins and ochratoxin A, the 2D-LC separation eliminated co-eluted interfering compounds, permitting quantification by solvent-only calibration standards. In contrast, matrix suppression was still observed for aflatoxins, requiring matrix matched calibration standards for quantitation.

Co-contamination of aflatoxins and other mycotoxins in foods and feeds is well known. How to efficiently monitor co-occurrence of aflatoxins and other mycotoxins has been a challenge for mycotoxin researchers [271,272,273]. When multiple mycotoxins are needed to be analyzed, it is clear that LC-MS would be a better analytical tool as it can achieve identification, quantitation and confirmation for multiple mycotoxins in one analysis, a desirable feature that other techniques lack. For example, the survey work performed by Lombaert et al. and Zhang et al. report mycotoxins in infant foods from the retail market, and provide a good comparison of the application of LC-MS-based multimycotoxin analysis vs. LC-FLD-based single mycotoxin analysis [274,275]. Without LC-MS, multiple LC-FLD methods had to be used to collect information of individual mycotoxins such as aflatoxins, deoxynivalenol and ochratoxin A, while using LC-MS, these mycotoxins could be identified and quantitated by one method. For multimycotoxin analysis, using multiple single mycotoxin methods would be much less practical than using a multimycotoxin method. As LC-MS could simultaneously detect a large number of mycotoxins in one analysis, the current trend is to develop multimycotoxin procedures, taking advantage of the analytical capacity of LC-MS, hence improving throughput. A variety of LC-MS-based multimycotoxin methods have been developed. In these methods, aflatoxins and other mycotoxins of interest are extracted from samples, followed by cleanup procedures, if necessary, and then analyzed by LC-MS.

In a series of studies [276,277,278,279,280,281], the number of mycotoxins that could be simultaneously detected in one LC-QQQ-MS analysis has been expanded from 39 to 300. Each mycotoxin is monitored by two unique MRM transitions and retention time, which is sufficient to achieve confident identification [282]. It is evident that for multimycotoxin analyses, the capacity of conventional TLC or LC-UV/FLD is unrivaled to modern LC-QQQ technologies. With increasing number of mycotoxins, researchers need to find appropriate extraction and sample clean up procedures for target mycotoxins with different physicochemical properties. Practically, it is also a QA/QC and logistical challenge to trace, store and prepare calibration standards for a large number of analytes.

Spanjer et al. [43] extracted 33 mycotoxins including aflatoxins in various products (peanuts, pistachios, wheat, maize, cornflakes, raisins and figs) using acetonitrile–water (80:20, *v*/*v*) and methanol-water (70:30, *v*/*v*), followed by dilution with water and LC-MS/MS analysis. Although matrix-matched calibration standards were prepared for quantitation, the practice is considered less laborious than using SPE or immunoaffinity columns for cleanup. Compared to LC-FLD methods, the LC-MS/MS method not only generated comparable results concerning aflatoxin B_1_ in FAPAs proficiency test samples but also quantitated other mycotoxins in one analysis. Mol et al. [283] proposed and evaluated a generic extraction approach using water-acetonitrile-1% formic acid as an extraction solvent to analyze 136 pesticides, 36 natural toxins (including aflatoxins) and 86 veterinary drugs in foods and feeds. After extraction, no further clean-up was performed, extracts were directly injected into an LC-QQQ. Recoveries for 80% of the analytes were between 70% and 120% with RSDs < 10%. Matrix effects were minimized by using matrix-matched calibration standards and a small injection volume, 5 μL. It is clear that with reasonable performance criteria, LC-MS-based multianalyte methods could significantly improve operation efficiency, replacing many matrix- and compound-dependent single mycotoxin methods [241,284,285].

The intended use of multimycotoxin methods should be clearly defined so that a practical balance between method performance and number of target mycotoxins could be reached. For example, Zhang et al. [55] developed a stable isotope dilution LC-MS/MS method to analyze aflatoxins, fumonisins, ochratoxin A, deoxynivalenol, HT-2/T-2 toxin and zearalenone in foods. As the method was intended for the FDA regulatory program, only FDA regulated mycotoxins (e.g., aflatoxins) and a few mycotoxins of health significance (zearalenone, HT-2/T-2 toxins) are included. Each mycotoxin was quantitated using a ^13^C-IS and the method could identify and quantitate these mycotoxins at concentrations ranging from 1.0 to 1000 ng/g in peanut butter, wheat flour and corn. The majority of recoveries ranged from 80 to 120% with RSDs < 20%. Greater than 90% of the average recoveries were in the range of 90–110%, with repeatability RSD_r_ (within laboratory) < 10% and reproducibility RSD_R_ (among laboratory) < 15%. Although such an approach would not be realistic for the quantification of 191 mycotoxins [279] or 295 bacterial and fungal metabolites in foods [284], a similar platform may be utilized for screening mycotoxin occurrence in different matrices.

To date, the majority of LC-MS-based mycotoxin methods have been developed using LC-QQQ, however, LC–high resolution mass spectrometry (HRMS; e.g., LC-Orbitrap-MS and LC-Q-time of flight-MS) has gradually attracted attention [285,286]. LC–HRMS offers the acquisition of high resolution full scan data with accurate *m/z* and the option of targeted or non-targeted sample acquisition with retrospective data analysis. Although it is still a niche application, LC–HRMS has been evolving rapidly in the field of mycotoxin analysis and provides a powerful tool to screen for aflatoxins and other mycotoxins in food and feed products. Early HRMS applications were stymied by the lack of impactful understanding and agreement among mycotoxin researchers regarding the role of LC–HRMS in mycotoxin analysis. The lack of software tools and identification criteria created severe technical impediments to the application of LC–HRMS, but rapid improvements of hardware (e.g., sensitivity, resolution power and data acquisition rate), data mining software and data storage systems supported by identification criteria guidance will likely facilitate the implementation of LC–HRMS moving forward [282,287,288].

Like LC-QQQ, LC–HRMS, especially LC-Orbitrap and LC-Q-Orbitrap MS is commonly used for multimycotoxin and multianalyte analysis. Due to limited use of LC-TOF in mycotoxin research, very few studies use LC-TOF or LC-Q-TOF for aflatoxin analysis. Toth et al. [289] used a LC-Q-TOF and studied fragmentation patterns of aflatoxins under different collision energies. No quantitative analysis was performed. In an early LC–HRMS study, Vaclavik et al. [290] coupled a direct analysis in real time (DART) ionization source with an Orbitrap MS for the quantitative analysis of multiple mycotoxins in wheat and maize. Sample extracts were directly analyzed by DART-Orbitrap MS without LC separation. The impact of resolution power (10,000–50,000 full width at half maximum, FWHM, at *m/z* 200) on quantitation and mass accuracy was evaluated. For aflatoxins, the sensitivity of the DART method was poor as compared to LC-QQQ. Lattanzio et al. [291] developed an LC–HRMS method for the determination of EU regulated mycotoxins (aflatoxins, ochratoxin A, fumonisins, deoxynivalenol and zearalenone) in cereals. A single stage LC-Orbitrap was operated in full-scan mode to monitor each mycotoxin by its molecular ion and fragments generated using high energy collision dissociation (HCD). At a resolving power of 100,000 FWHM, mass accuracy of targeted mycotoxins ranged from 0.1 to 3.9 ppm. In terms of sensitivity (e.g., LODs), recoveries and repeatability, the method offered analogous performance as compared to a previously validated LC-QQQ method. Later, Liao et al. [57,262] compared quantitation on an LC-Q-Orbitrap operated in full scan and data-dependent acquisition mode, to an LC-QQQ and concluded that both LC-MS methods had sufficient sensitivity to identify and quantitate multiple mycotoxins, albeit different identification criteria were used. Using an LC-Q-Orbitrap MS, Renaud and Sumarah [292] developed an LC-data independent acquisition method. In this approach, both quantitative and qualitative analyses could be conducted using collected data. For mycotoxins including aflatoxins with available standards, they were identified and quantitated similar to conventional LC-MS analyses. Using a similar approach, Castaldo et al. [293] developed an LC-Q-Orbitrap method, which combined target analysis and non-target screening of mycotoxins in pet food. With better resolution power, data acquisition rates and accurate mass spectra libraries, this approach is considered as a more advanced and efficient method for mycotoxin analysis.

This review focuses on aflatoxins but not multimycotoxin analysis, so the above discussion aims to use a few representative studies to illustrate major trends and challenges concerning LC-MS based mycotoxin analysis and the impact of LC-MS on aflatoxin analysis. In summary, the introduction of LC-QQQ-MS has transformed the conventional single/single class-mycotoxin-based approach to multimycotoxin identification and quantitation. LC–HRMS enables both targeted and non-target data analysis, leading to a new mycotoxin testing paradigm. As aflatoxin analysis is a multifaced challenge, existing sampling plans, sample preparation techniques and method performance criteria (validation) should be carefully evaluated and revised if necessary so that the potential of LC-MS-based technologies could be fully reached in the coming years to increase the sensitivity, accuracy, specificity and efficiency of aflatoxin analysis. Noteworthy, rapid assays (e.g., ELISA) and LC-FLD methods are expected to compete with LC-MS, as there are substantial economic interests associated with mycotoxin analysis in the industrial market [286], stymying the existing efforts to increase the use of LC-MS that has been gaining increasing popularity among mycotoxin laboratories. Several studies compared LC-MS/MS to other analytical techniques used for aflatoxin analysis, suggesting LC-MS/MS generated comparable results compared to ELISA and LC-FLD in terms of precision and accuracy [294,295,296]. The choice of a method depends on many factors, including but not limited to data quality of quantitation. If mycotoxin molecular identification is required, LC-MS has been shown to be the method of choice.

### 3.4. Gas Chromatography

Aflatoxins are semi-volatile compounds permitting analysis by GC. Unfortunately, GC-based aflatoxin analysis has never achieved wide attention. When capillary columns were not available, one could not use GC to analyze aflatoxins due to insufficient separation of aflatoxin B_1_, B_2_, G_1_ and G_2_ on pack columns. The advent of capillary columns, however, encouraged researchers to explore this chromatographic tool for aflatoxin analysis. In 1981, Friedli [297] developed a GC–MS method for the determination of aflatoxin B_1_, in which aflatoxin B_1_ was introduced into the GC via on-column injection, followed by the separation on a fused silica capillary column coated with a nonpolar phase (SE-30) and detection using MS. Without chemical derivatization, experiments required an on-column mass of 10 ng for detection and no food samples were analyzed. Two similar studies were reported by Trucksess et al. [251] and Rosen et al. [298], in which aflatoxins were determined by the MS detector coupled with the GC but separated on different GC capillary columns. Goto et al. [299,300] reported the analysis of aflatoxins using GC-FID. In these studies, GC–MS and GC-FID were manly evaluated as a qualitative tool for the confirmation of aflatoxins in suspected samples. Due to the lack the simplicity of TLC and the sensitivity of LC-FLD, GC-based aflatoxin analysis has not been extensively evaluated compared to TLC and LC-FLD. Currently, given the increasing use of LC-MS for mycotoxin analysis, it is difficult to see that there will be a resurgent interest in GC as there are already many LC-FLD and LC-MS methods developed for the determination of aflatoxins in various food matrices.

### 3.5. Capillary Electrophoresis

As an electrokinetic separation technique, capillary electrophoresis is performed in submillimeter diameter capillaries or micro- and nanofluidic channels filled with a buffer solution. In an electric field, charged analytes and matrix components in the buffer would migrate with different rates depending on their charges and sizes, hence being separated. Benefits of CE include good separation efficiency (separation achieved within 1 min), ease in the instrument setup and operation and small sample and buffer volumes (nL ranges). Flexible separation conditions can be optimized by changing buffer additives. Conventional capillary zone electrophoresis CZE and micellar electrokinetic capillary chromatography (MECC) are the two major techniques that have been evaluated for aflatoxin analysis [301,302]. Advances in microfabrication techniques developed in the semiconductor industry enable the miniaturization of CE on microchips [303]. Since the 2000s, there has been increasing attention to capillary CE chip-based mycotoxin analysis. Coupled with modern MS, these CE chips could lead to innovative applications for aflatoxin analysis [304].

Holland and Sepaniak [305] used MECC to separate 10 mycotoxins, including aflatoxin B_1_, B_2_, G_1_ and G_2_ but the study focused on separation capacity of the system and how to normalize retention times of target analytes with no quantitative data provided. Cole et al. [306] carefully evaluated the key parameters (e.g., internal diameters of capillaries, viscosity and compositions of mobile buffer and fluorescence properties for aflatoxins in various solvents) that could affect micellar electrokinetic capillary chromatography for aflatoxin analysis. The separation of aflatoxin B_1_, B_2_, G_1_ and G_2_ was achieved within 30 s using a capillary column (40 cm L × 25 μm ID) and detected using an on-column laser-based fluorescence detection. The limits of detection are 1000 ppb and extensive SPE cleanup was performed prior to the injection of corn sample extracts. Sensitivity of the method, however, is insufficient to detect aflatoxins at or below the target U.S. regulatory limit of 20 ppb. In a later study, Maragos and Greer [307] achieved a much lower limit of detection (0.5 ppb) of aflatoxin B1 in corn by CE and FLD. Average recoveries of aflatoxin B_1_ spiked at 1–50 ppb are around 85%. Due to the very low on-column mass of CE and fluorescence interference from the CE buffer or matrix components, these studies suggest the need of more specific and sensitive detection technologies. Jiang et al. [308] used integrated plastic microfluidic devices with ESI-MS to separate and detect aflatoxins. In the study, an in-house microfabricated affinity dialysis and concentration system was developed. Specific detection of aflatoxin B_1_ was achieved by optimizing the molar binding ratios of aflatoxin B_1_ antibody to each aflatoxin and a flow rate around 100 nL/min. The study shows potential of miniaturization of CE but follow-up studies are needed to demonstrate its applicability in foods for aflatoxin analysis. Furthermore, when CE is coupled with MS, cost and complexity of the system is no less than that of LC-MS. Between a well-established technique (LC-MS) and an analytical technique with certain potentials (CE-MS) for aflatoxin analysis, preference is most commonly given to LC-MS-based technologies. It is expected that CE especially CE microchips will remain as a research-oriented technique in the near future.

## 4. Conclusions

Aflatoxin contamination will remain a global public health concern yet challenges that each country/region faces are very diverse. Global trading, climate changes and diverse regulatory policies have further complicated the issue. Therefore, the need for analytical tools to combat mycotoxin contamination varies, prompting the development of broad ranges of chromatographic and mass spectrometric methods for the determination of aflatoxins. This review highlights trends and novel techniques in the course of the history of aflatoxin methodology, with emphasis on applications of sample preparation, chromatography, and mass spectrometry to food analysis. Aflatoxins are heterogeneously distributed in foods and feeds so each step including sampling, sample preparation, extraction, clean-up and instrumental determination plays a crucial role in the analysis of aflatoxins. The trend is obvious any new techniques can outperform existing ones in terms of specificity, sensitivity and throughput will be appreciated. Following the transition from TLC, LC-UV/FLD to LC-MS, advances in detection technologies are complimented by the simplification of sample preparation, transforming multistep extraction/clean-up to one step “dilute-and-shoot”. Similarly, identification has shifted from non-specific properties (e.g., visual inspection, UV adsorption or fluorescence emission) to intrinsic properties (e.g., *m/z*, ion ratios, molecular fragmentation) and traditional single-/single-class mycotoxin analysis has been gradually replaced by multimycotoxin analysis using LC-MS. In the future development of analytical methods, these are the areas where research will focus on. Our ability to replace and upgrade current techniques in these areas will, to a great measure, determine our success to monitor aflatoxins in foods.
